# Translating innovation in biomedical research: Design and delivery of a competency-based regulatory science course

**DOI:** 10.1017/cts.2019.432

**Published:** 2019-12-23

**Authors:** Alexandra J. Greenberg-Worisek, Katherine E. Cornelius, Luz Cumba Garcia, Felicity T. Enders, Nilay D. Shah, Anthony J. Windebank

**Affiliations:** 1 Center for Clinical and Translational Science, Mayo Clinic, Rochester, MN, USA; 2 Department of Health Sciences Research, Mayo Clinic, Rochester, MN, USA; 3 Yale-Mayo Center for Excellence in Regulatory Science and Innovation, Mayo Clinic, Rochester, MN, USA; 4 Mayo Clinic Graduate School of Biomedical Sciences, Mayo Clinic, Rochester, MN, USA

**Keywords:** Regulatory science, translational science, graduate biomedical research education, FDA

## Abstract

As the pace of biomedical innovation rapidly evolves, there is a need to train researchers to understand regulatory science challenges associated with clinical translation. We describe a pilot course aimed at addressing this need delivered jointly through the Mayo Clinic Center for Clinical and Translational Science and the Yale-Mayo Center for Excellence in Regulatory Science and Innovation. Course design was informed by the Association for Clinical and Translational Science’s Regulatory Science Working Group’s competencies. The course used didactic, case-, and problem-based learning sessions to expose students to regulatory science concepts. Course evaluation focused on student satisfaction and learning. A total of 25 students enrolled in the first two course deliveries. Students represented several disciplines and career stages, from predoctoral to faculty. Students reported learning “an incredible amount” (7/19, 36.8%) or “a lot” (9/19, 47.4%); this was reflected in individual coursework and their course evaluations. Qualitative feedback indicated that assignments that challenged them to apply the content to their own research were appreciated. The heterogeneity of students enrolled, coupled with assessments and course evaluations, supports the statement that there is a growing need and desire for regulatory science-focused curricula. Future research will determine the long-term impact.

## Introduction

We are now living in a time of incredible innovation, with many new biomedical technologies, such as CRISPR, stem cells, and additive manufacturing, giving hope to patients and clinicians. Many providers and researchers working at academic medical institutions are eager to translate novel therapies into first-in-human trials and are partnering with the private sector in preparation for doing so. However, the rapid evolution of these technologies is outpacing the ability of regulatory scientists at the US Food and Drug Administration (FDA) and international regulatory agencies. To determine tests, metrics, and standards for ensuring reproducible high-quality, safety, and efficaciousness of products and therapies made through application of these new methods. Having observed the effects of treatments based on newer technologies that were translated before they were fully understood, there is understandably hesitance from regulators to give the green light without additional study [1,2].

In parallel with these exciting technological developments is the rise of the field of regulatory science. While often conflated and overlapping in some areas, regulatory *affairs* and regulatory *science* are distinct but complementary fields. Regulatory affairs are defined by the Organization for Professionals in Regulatory Affairs as “controlling the safety and efficacy of products in areas including pharmaceuticals, veterinary medicines, medical devices, pesticides, agrochemicals, cosmetics, and complementary medicines”, that is, the process of ensuring that products meet regulatory standards for safety, efficacy, and quality [3]. Regulatory science, on the other hand, is defined by the FDA as “the scientific and technical foundations upon which regulations are based in various industries – particularly those involving health or safety” [4]. In a landmark 2011 document, the FDA defined eight specific priority areas for regulatory science; these included “Modernizing Toxicology to Enhance Product Safety” and “Stimulate Innovation in Clinical Evaluations and Personalized Medicine to Improve Product Development and Patient Outcomes,” among others. Each year, these priority areas are updated and/or added to as technologies continue to evolve.

In 2012, the FDA began to fund Centers for Excellence in Regulatory Science and Innovation, or CERSIs, in an effort to tap into scientific expertise at other institutions to try and more quickly find the best way to assess the safety, efficacy, and quality [5]. The ultimate hope is that, by engaging more minds at leading institutions in addressing these challenges, innovations can be translated into clinical practice more quickly while still protecting the public health. There are currently four FDA-funded CERSIs, including Johns Hopkins, University of California San Francisco-Stanford, University of Maryland, and Yale University-Mayo Clinic.

In addition to contributing to regulatory science research, CERSIs are also charged with innovating and delivering regulatory science training at all levels, from predoctoral students to faculty-level clinicians [5]. Separately, the Association of Clinical and Translational Science (ACTS) established a Regulatory Science Working Group to address the need for regulatory science education in Clinical and Translational Science Awardee (CTSA) institutions [6]. The ACTS Regulatory Science Working Group has previously published a comprehensive set of themes and competencies which students working at the intersection of translational and regulatory science must be aware of and/or develop expertise in as they become leaders in this space [7].

Recognizing this growing need, the Yale-Mayo CERSI and the Mayo Clinic Center for Clinical and Translational Science (CCaTS) worked collaboratively to design and deliver a new course aimed at introducing concepts of regulatory science to students in both the Yale-Mayo CERSI Students program and the CCaTS certificate, master’s, and doctoral programs. This course, based upon the aforementioned published competencies and inspired by a didactic course created at Georgetown University [8], uses a blended format of didactic lectures, case-based learning, and problem-based learning to expose students to regulatory science concepts [8]. More importantly, the course is designed to trigger students to thinking about how they might work to develop quality and safety assessments and metrics that would apply to their own laboratory work as they pursue research with these cutting-edge technologies. Here, we detail the design and initial deliveries of this foundational course, including details about student assessment and student course evaluation.

## Methods

The course detailed here, *Introduction to Regulatory Science*, is the second in a planned 3-course series in Regulatory Science and Affairs and is a collaborative effort between Mayo Clinic CCaTS and the Yale-Mayo CERSI (Table 1). Briefly, the first course, *Regulatory Issues in Clinical Research*, is a high-level regulatory affairs survey course that exposes students to key points of oversight and regulatory bodies involved across the translational spectrum, from wet-lab and animal studies through to population health and entrepreneurship [9]. It is delivered in a blended format and affords students the opportunity to participate in a Mayo Clinic Institutional Review Board session, including reviewing and presenting an assigned protocol to the committee. It is a required core course for all CCaTS certificate and degree programs and was designed to meet competencies set forth for CTSA programs by the National Center for Advancing Translational Science [10]. *Case Studies in Regulatory Science* is the upcoming third course in the series and builds upon the success of our other case-based course offerings in translational science, individualized medicine, and entrepreneurship [11].

**Table 1.** Sample schedule for Introduction to Regulatory Science, including an overview of course sessions, objectives, and assignments

Week	Session topic	Session objectives	Assignments
1	Introduction	Define regulatory science	Quiz
		Distinguish between regulatory science and regulatory affairs	
		Explain the importance of regulatory science throughout the clinical translation process	
2	FDA Regulation: a historical perspective	Describe the history of the FDA as a regulatory body and explain the reason behind its establishment	Quiz
		Summarize the evolution of product regulation over time	
		Compare and contrast regulatory affairs and regulatory science	
		Illustrate how regulatory affairs and regulatory science intersect in practice using specific examples	
3	The “Product Review Lifecycle”	Explain the key points during the product development process at which regulatory oversight is required	Quiz
		Compare and contrast the review cycle processes for drugs, biologics, and devices	
		Illustrate the differences between the different special review pathways and designations	
		Hypothesize how a given new technology or test could impact the product review process	
4	The FDA’s Eight Priority Areas for Advancing Regulatory Science	Describe the FDA’s eight priority areas for advancing regulatory science	Quiz
		Illustrate the use of regulatory science in a product review case study	
		Explain FDA initiatives to increase innovation in regulatory science, including their CERSI program	
		Using real-world data provided, illustrate the need for innovation in regulatory science in the USA	
5	Understanding the role of the Advisory Committees and Meetings	Explain the FDA Advisory Committee system	No Quiz
		Compare and contrast the roles of Advisory Committees in the review of drugs, biologics, and devices	
		Interpret and evaluate information and materials presented during an FDA Advisory Committee meeting	
6	Innovations in the science and conduct of clinical trials	Describe the standard view of clinical trials	Quiz
		Illustrate challenges in the design and conduct of clinical trials using examples	
		Discuss specific new techniques in clinical trials and the science underlying their design	
		Examples have included: “n of 1” trials, adaptive trial design	
7	The role of bioethics in regulation	Describe the different ethical viewpoints regarding use of human embryos in stem cell research	Quiz
		Identify challenges in governance of new technologies using stem cell research and practice as a case study	
		Compare and contrast the responsibilities and roles of doctors, policymakers, professional societies, and international bodies in regulating the clinical translation of emerging stem cell therapies	
8	Toxicology and product safety	Describe the purpose of preclinical safety evaluations	Quiz
		Summarize the types of preclinical safety studies required by FDA	Final paper proposal due
		Discuss the problem of choosing a relevant species for preclinical testing and illustrate with examples	
		Explain GLPs and their importance in preclinical toxicology and safety testing	
		Generate a plan to incorporate GLP, safety, and toxicology principles into preclinical studies for your research	
9	Evaluating Emerging Technologies	Describe what an “emerging technology” is in the eyes of regulators	Quiz
		Summarize the FDA’s proposed steps to evaluate emerging technologies	
		Explain the general process of the creation of an FDA guidance document for a new technology	
		Analyze emerging technologies for key factors that may affect quality, safety, and efficacy of a product for which assessment testing may be necessary	
		Develop ideas for assessment of identified concerns with regard to an emerging technology	
		Examples have included 3D printing and tissue printing	
10	Innovations in bioinformatics for regulatory oversight	Summarize the strengths and weaknesses associated with secondary data analysis techniques	Quiz
		Describe the role of data mining and big data in regulatory science, including in postmarket surveillance	
		Compare and contrast existing standards of big data research and reporting	
		One prior example presented used secondary data mined from multiple sources to monitor opioid prescriptions	
11	Product quality	Identify the FDA requirements that apply to regulated products	Quiz
		Compare and contrast the similarities/differences in requirements	
		Explain the importance of a Quality Management System	
		Describe the consequences of noncompliance with federal requirements	
12	Product manufacturing	Identify the FDA requirements that apply to regulated manufactured products	Final Paper Due
		Understand the elements that constitute a SOP and a batch record	
		Discuss the concept of data integrity	
		Emphasize the importance of risk management	

CERSIs, Centers for Excellence in Regulatory Science and Innovation; GLPs, Good Laboratory Practices; SOP, standard operating procedure.

### Course Design

The primary objective of *Introduction to Regulatory Science* is a 3-month course (12 contact hours over 12 weeks) that aims to provide students exposure to regulatory science concepts and methods through a combination of didactic, case-based, and problem-based learning sessions. While this course offering is viewed as a second in our “series” after a regulatory affairs course (Table 1), it is designed to serve as a stand-alone course and has no prerequisites, allowing anyone at Mayo Clinic with an interest in regulatory science to enroll.

#### Course objectives and content

Course objectives were developed based upon the ACTS Regulatory Science Working Group priority areas [7]. The course objectives for *Introduction to Regulatory Science* are as follows:

- Define “regulatory science.”
- Recognize differences between “regulatory science” and “regulatory affairs.”
- Explain each of the eight priority areas identified by the FDA for advancing regulatory science.
- Determine what bioethical and safety concerns need to be considered and addressed by regulatory science tools in a case study.
- Summarize what regulatory standards, tools, and approaches are used in a given case study, and evaluate whether the underlying science supports the use of those tools.

As the course is designed to be taken alone or in concert with *Regulatory Issues in Clinical Research*, an introductory lecture is provided early in the course regarding drug, biologic, and device approval processes to ensure all participants understand the vernacular and processes which regulatory science research aims to support and innovate. Weekly topics are drawn from the FDA’s “Advancing Regulatory Science” report’s Priority Areas; these include toxicology and product safety, innovations in the science and conduct of clinical trials, product manufacturing and quality, evaluating emerging technologies, using informatics to improve health outcomes [4]. While content within each broad topic area may change from year to year to reflect the quickly evolving science and therapeutic discoveries, the core themes remain the same. Additionally, a session exploring the role of advisory committees and how regulatory science is applied during such review panels is included, in which students are asked to review the submission information packets and vote on the key questions surrounding methodology; this is done utilizing publicly available video clips. This exercise was adapted from a course delivered at Harvard T.H. Chan School of Public Health, “*Statistical and Quantitative Methods for Pharmaceutical Regulatory Science*,” taught by Dr. Marcia Testa and Dr. Robert O’Neill (BST 217/BIO 523) [12].

#### Target audience

The target audience for *Introduction to Regulatory Science* is heterogeneous, as it is offered as an elective and is open to both degree-seeking and nondegree-seeking students and trainees across the Mayo Clinic enterprise through the Mayo Clinic Graduate School of Biomedical Science (MCGSBS). Potential students include predoctoral trainees, postdoctoral fellows, CCaTS Master’s and Certificate students, KL2 students, medical students, and clinical residents and fellows. Additionally, faculty and staff (including Allied Health) are eligible to take the course if interested. To help facilitate deep discussion during class sessions, the course is capped at 20 students per delivery. Students from remote Mayo Clinic sites (Mayo Clinic Health System sites, Mayo Clinic Arizona, and Mayo Clinic Florida) are eligible to participate.

### Course Delivery

The course was first delivered at MCGSBS during the Summer quarter of the 2017–2018 Academic Year; it has since been delivered a second time during Summer quarter of the 2018–2019 Academic Year.

### Online Course Site

To deliver course materials for each session, the Blackboard Learning Management System was used. In addition to recommended readings and lecture slides, each week’s Blackboard module also contained an open-note quiz serving as a knowledge/comprehension check. Students were not allowed to collaborate on these assessments, and they had to be completed in one sitting. Instructions and rubrics for the final paper were also included. The assignments were submitted through Blackboard for grading using the corresponding assignment rubrics and were graded blind to ensure fairness in student assessment.

#### In-person sessions

Course sessions were held weekly for 1 hour, each over the span of an academic quarter (12 weeks). Students were expected to attend all sessions. For students located in Arizona or Florida, the course was webcast live to reserved rooms at their respective sites; students at these locations had microphone capabilities, allowing them to actively participate in discussion and ask questions to the lecturers. No major issues arose that were reported by those off-site.

As most course sessions focused on independent Priority Areas and did not build upon other Priority Areas, the order of lectures was largely dependent upon speaker availability. Many of our speakers did not consider themselves “regulatory science experts,” *per se*, but were identified as researchers who were working in the priority area space without having necessarily labeled themselves as such. Lecturers were identified by approaching leaders in departments related to priority areas (i.e., molecular pharmacology group for the Toxicology-focused lecture) and asking for speaker recommendations and volunteers. Across both course deliveries, however, the introductory and regulatory affairs-focused lectures were included at the beginning of the course to ensure all students had same basic vocabulary and understanding.

During the second course delivery (2018–2019 academic year), a teaching assistant was added to the course team; this individual had taken the class the prior year and served as a liaison between the course directors and the guest lecturers and provided guidance to students on their final papers via optional one-on-one meetings, as requested.

### Assessment of Students

Students were assessed based upon course attendance and participation, weekly Blackboard quizzes, a final paper proposal, and the final paper. Detailed instructions and rubrics were provided to the students in advance of their paper proposal and their final paper to ensure transparency in grading; the hope was that this would alleviate concerns about grading and allow students to focus on the content (see Supplementary Materials for documents).

The paper proposal and final paper aimed to encourage students to apply knowledge of regulatory science learned throughout the course to their personal research or a case close to their area of interest. The papers had a page limit of 5–7 pages, double-spaced, and required that students included: (1) an overview of the discovery, product, or treatment, including indications and citing any published trials prior to approval (if applicable); (2) a discussion of safety, efficacy, and quality considerations that may have been or are associated with the discovery, product, or treatment; (3) any existing FDA regulations or guidances that might apply and, if none are available, what would need to be created to provide guidance; and (4) a discussion of the science that supports the identified regulations and guidances. If none are available, the student is asked to propose guidelines that the current literature would support. Students are specifically asked to identify 1–3 FDA regulatory science priority areas which may apply to their case. While writing their final papers, students were highly encouraged to use MedDATA Foundation’s Free FDA Information Repository (FDA IRAI, irai-online.org), a collaboration between the MedDATA Foundation and the FDA, which provides enhanced “searchability” of FDA’s primary information resources and training materials for students and educators in the regulatory science field.

### Student Course Evaluation

Upon completion of the course, students were asked to complete the standard end-of-course satisfaction survey used throughout CCaTS. Briefly, this course survey includes both quantitative and qualitative items that address satisfaction of course content, course directors, and online resources; additionally, it asks students to rate how much they felt they gained out of the course and whether the course was worth their efforts. Surveys are kept completely anonymous, gathered by the Mayo Clinic Survey Research Center, and shared as a single report with course directors approximately 1 month after course completion.

### Statistical Analyses

Descriptive statistics were conducted using quantitative data from the end-of-course satisfaction survey. Specifically, evaluation of the course focused on whether students felt the course objectives were met, the interactive nature of the course supported learning, and the course worth the effort invested in it. Additionally, we reviewed students’ final papers to determine the most frequently cited FDA Regulatory Science Priority Areas they felt relevant to their work.

## Results

### Student Characteristics

Students enrolled in both the 2017–2018 and 2018–2019 course deliveries were heterogeneous in terms of location, position at Mayo Clinic, career stage, and specialty (Table 2). Predoctoral and master’s trainees represented three of the MCGSBS’ seven PhD tracks, including clinical and translational science, immunology, and molecular pharmacology and experimental therapeutics. Clinical specialties represented by residents, fellows, and faculty included preventive medicine, obstetrics and gynecology, cardiology, radiology, nephrology, clinical genomics, and surgery. The majority of students in both years were based in Rochester (88%) and physically attended class, most also enrolled for credit (84%). In the first year of the course (2017–2018), students were mostly predoctoral trainees (37.5%) and allied health staff (62.5%); in the second year (2018–2019), the distribution was split between predoctoral trainees (29.4%) and residents/fellows (41.2%).

**Table 2.** Student characteristics for those participating in the 2017 and 2018 deliveries of CTSC 5025: Introduction to Regulatory Science at Mayo Clinic’s Center for Clinical and Translational Science

Course delivery	2017, *n* (%)	2018, *n* (%)
Total number of students enrolled	8	17
Number of auditors	3 (37.5)	1 (5.9)
Student/Auditor location		
MCR	7 (87.5)	16 (94.1)
MCA	1 (12.5)	1 (5.9)
MCF	0	1 (5.9)
Student position		
Predoctoral trainee	3 (37.5)	5 (29.4)
Master’s	1 (12.5)	2 (11.8)
Postdoctoral fellow	1 (12.5)	3 (17.6)
Resident/Fellow	1 (12.5)	7 (41.2)
Employee (faculty of allied health)	5 (62.5)	1 (5.9)
Survey response rate	7/8 (87.5)	12/17 (70.6)

MCA, Mayo Clinic Arizona; MCR, Mayo Clinic Rochester (Minnesota); MCF, Mayo Clinic Florida.

### Student Final Assessments

**Fig. 1.**
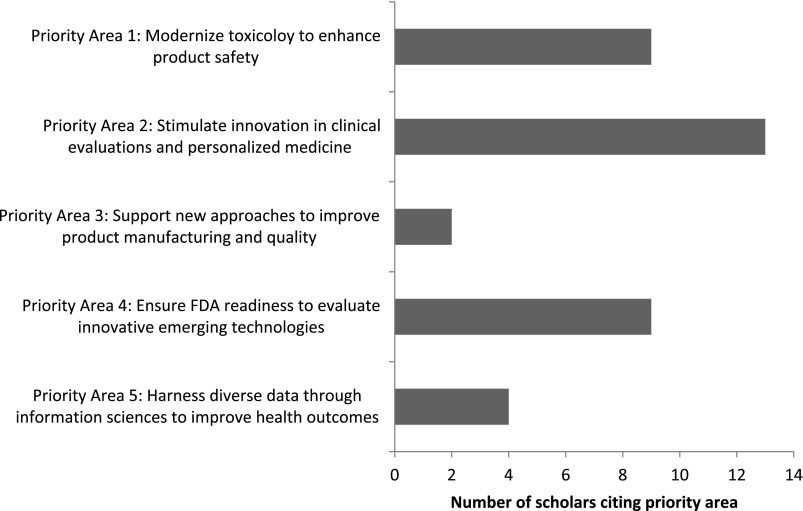
Distribution of FDA Regulatory Science Priority Areas selected as applicable to personal areas of work among scholars participating in CTSC 5025: Introduction to Regulatory Science at Mayo Clinic Graduate School of Biomedical Science in 2017–2019 (*N* = 37 citations across 25 scholars).

Students’ final case study papers encompassed a wide variety of topics, reflective of the diversity in specialties and disciplines represented by the two cohorts. Students were evenly split between writing their final papers about a publicly available case and their own personal research. This was quantified by reviewing students’ final papers to identify which FDA Regulatory Science Priority Areas were cited (Fig. 1). While students were allowed to cite multiple priority areas, most selected only one. The most frequently cited priority areas were “Priority Area 2: Stimulate Innovation in Clinical Evaluations and Personalized Medicine” (13/37, 35.1%); this was followed by “Priority Area 1: Modernize Toxicology to Enhance Product Safety” (9/35, 24.3%) and “Priority Area 4: Ensure FDA Readiness to Evaluate Innovative Emerging Technologies” (9/35, 24.3%). The other two priority areas cited were “Priority Area 3: Support New Approaches to Improve Product Manufacturing and Quality” (2/37, 5.4%) and “Priority Area 5: Harness Diverse Data through Information Sciences to Improve Health Outcomes” (4/37, 10.8%); the other six priority areas identified in the FDA’s Strategic Plan were not cited.

### Student Course Evaluations

**Fig. 2.**
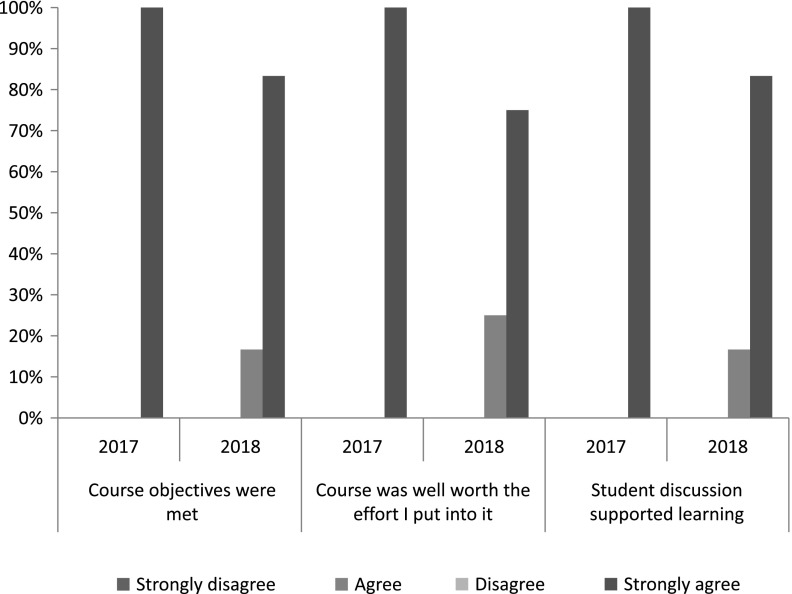
Distribution of key items on postcourse scholar satisfaction evaluation among scholars participating in CTSC 5025: Introduction to Regulatory Science at Mayo Clinic Graduate School of Biomedical Science in 2017–2019 (*N*_2017–2018_ = 7, *N*_2018–2019_ = 12).

Survey response rates were high for both course deliveries (87.5% [7/8] in 2017–2018, 70.6% [12/17] 2018–2019; Table 2). All students across both class years agreed or strongly agreed that course objectives were met (Fig. 2). Additionally, 84.2% (16/19) of students over the 2 years strongly agreed that the course was “well worth the effort I put into it.” Respondents also reported learning “an incredible amount” (7/19, 36.8%) or “a lot” (9/19, 47.4%); no students reported that they learned “nothing” (Fig. 2). Regarding the interactive course design, 100% of students in 2017–2018 and 83.3% of students in 2018–2019 strongly agreed that “discussion supported learning” (Fig. 2).

Qualitative feedback was generally positive, indicating that the team-taught and in-person course design choices were highlights for trainees. Notable comments included:

- “Appreciated all the experts presenting on their topics which was an excellent way to be exposed to thought leaders & investigators at Mayo. The course was very well organized.”
- “…classes, despite the dry content, are dynamic, interesting, and organized, all of which are difficult to obtain simultaneously.”
- “The course was very informative. There were many experts who gave presentations which were very diverse.”
- “Wasn’t as relevant to my interests as I had thought – I learned a good deal, but will not likely retain much due to that disparity between what was taught and what I will use. I probably wouldn’t take it again if given the choice, but was glad to have the introduction at the least.”

Cumulatively, 84.2% (16/19) of students gave the course a grade of “A” and 15.8% (3/19) gave it a “B.”

## Discussion

Our overarching objective was to design, deliver, and iterate a regulatory science-focused curriculum aimed at exposing a heterogeneous group of students to regulatory science concepts and case studies. The ultimate goal of doing so was to challenge trainees and researchers to think about how best to develop safety, efficacy, and quality metrics and tests for their own innovative areas of research. For the introductory course detailed here, *Introduction to Regulatory Science*, we sought to accomplish this through a blend of traditional lectures, as well as case- and problem-based learning [8].

Overall, student assessments demonstrated clear understanding of the content and the beginnings of exploring how regulatory science might apply to their personal research interests. Both student assessments and course evaluation indicate that the course objectives were met and that the course helped students’ progress toward the ACTS Regulatory Science Working Group competencies [7]. There was diversity in students enrolled, both in terms of research discipline as well as career stage. Furthermore, several students enrolled were not in CCaTS programs. We believe that this speaks to the demand for such content across biomedical research disciplines, and the need for such training programs to have a broader reach beyond those in translational science, regulatory science, and regulatory affairs programs.

Strengths of this course included competency-based design, the expertise of the faculty lecturers from within Mayo Clinic CCaTS and the Yale-Mayo CERSI, the breadth of the course content, and the small class size, which afforded rich discussion and peer-to-peer learning. While this class was a survey course, we sought to challenge our students to do more than memorize content by designing the course objectives and assignments to cross the various levels of Bloom’s taxonomy [13]. An example of this was in the design of the final paper, which challenged students to apply the content learned in class to their own research programs; many students reported in their qualitative that this exercise was “challenging, but [writing the paper] helped me understand the FDA world better” and was more beneficial than a “typical” exam.

While the small class size allowed for productive and fruitful discussions, it does present as a limitation of our findings in that the course was offered as an elective; therefore, only a self-selected group of students with particular interest in regulatory science participated. However, the enrollment in the first year was on par with the enrollment in most elective courses delivered in CCaTS (around 6–10 students). For the second delivery, enrollment almost doubled suggesting word-of-mouth validation that “I would recommend this course to other learners”. A second challenge, as noted in the qualitative feedback received, is that some students thought the term “regulatory science” was synonymous with “regulatory affairs” and therefore did not learn the information they had anticipated from the course. We are making concerted efforts to clarify this moving forward with our course marketing. A third limitation is that, due to changes in the rapidly evolving field of regulatory science, coupled with guest lecturer availability, the content presented for each priority area topic changed slightly between the deliveries to ensure that students were receiving the most up-to-date information. Finally, we focused our efforts on student satisfaction/reaction and learning – the first two levels of Kirkpatrick’s Four Levels of Learning Evaluation [14]. Given the time it takes to translate findings and move discoveries from laboratory into first-in-human trial, we have not yet had the opportunity to evaluate student behavior (application of learned content) and results (outcomes as a result of application of learned content) and do not yet know the long-term impact of this coursework on students’ research.

In the present report, we shared details regarding the design, development, and delivery of a new Regulatory Science course offered as part of CCaTS and the Yale-Mayo CERSI programs. Future development of this curriculum includes piloting the third course in the series, *Case Studies in Regulatory Science*, and continuing to iterate on *the current course* as the field of regulatory science continues to evolve. We are planning follow-up surveys with students from the initial two cohorts to assess student behavior and results (the two higher levels of Kirkpatrick’s model); this will also include items inquiring about what content they feel was missing after moving forward with translating their research and navigating the related regulatory science challenges. Finally, we will use the information gleaned from these follow-up surveys to report back to the ACTS Regulatory Science working group to inform future revision of the Regulatory Science competencies.

## Supplementary material

For supplementary material accompanying this paper visit http://dx.doi.org/10.1017/cts.2019.432. click here to view supplementary material
