# Supplementary material for: Translating innovation in biomedical research: Design and delivery of a competency-based regulatory science course
Source: J Clin Transl Sci. 2019 Dec 23;4(1):8–15. doi: 10.1017/cts.2019.432 (PMC7103473; doi:10.1017/cts.2019.432)
Supplement: Supplementary file 1 [file S2059866119004321sup001.docx]

CTSC 5025: Introduction to Regulatory Science

Summer 2017

# Regulatory Science Case Study Final Paper

For the final project in CTSC 5025, you will select a new or recently reviewed technology or product for which scientific FDA oversight would be necessary. Students are expected to develop their case study of choice into a final paper. The goals of this final paper are to:

1. Provide a brief scientific primer of the technology product (background, key preclinical studies and early trials, etc.)
2. Highlight regulatory science concerns related to their case study (safety, efficacy, and/or quality-related)
3. Discuss the scientific recommendations which were made to address these concerns

### Final Paper Timeline

| **Task** | **Due Date** | **Percentage of Final Grade** | **Additional Comments** |
| --- | --- | --- | --- |
| Paper Proposal | Thursday, August 24^th^, 2017 at 9am | 10% | - Proposals must be uploaded to Blackboard by the beginning of lecture on Thursday, August 24^th^, 2017 - Instructors will review paper proposals and provide feedback in approximately one week via email (By Friday, September 1^st^, 2017) |
| Final Paper | Thursday, September 21^st^, 2017 at 9am | 25% | - Final papers must be uploaded to Blackboard by the beginning of lecture on Thursday, September 21^st^, 2017 (Last day of class) |

*Grading rubrics for both the paper proposal and final paper are available through the course Blackboard site.*

### Paper Proposal Instructions

A 1-2 paragraph proposal (no longer than 2 double-spaced pages) of the case study/topic will be due by the beginning of Lecture 7 (August 24^th^, 2017). This proposal should:

- Identify a product, technology, or other regulatory science topic of interest
- Define some of the associated safety, efficacy, and quality concerns associated with the selected topic
- Identify a relevant clinical trial, guidance, or other regulatory document associated with the product or technology
- Name the regulatory science priority area with which the topic is associated
- Identify associated regulatory pathways
- Include citations as appropriate, including citations for guidances and reports

In selecting your case, we recommend searching the FDA website for recent workshops and advisory committee meetings and selecting a case for which the FDA has published resources (i.e., briefing package, webcast, PowerPoint slides, reviews, etc).

###

### Final Paper Instructions

Students will use their paper proposal and instructor feedback to develop their topic into a final case study paper. The paper should be 5-7 pages in length, double-spaced (not including references). References should be formatted into a bibliography at the end of the paper using the JAMA citation style; these should include primary research publications, guidances, and relevant committee meeting resources, as appropriate.

The final paper should include the following:

- A brief scientific primer of the case (biology/background, key preclinical studies and early trials, etc.)
- 2-3 safety, efficacy, and/or quality considerations that were or may have been/are associated with the case
- FDA guidances, regulations, and/or processes which might apply to the case
- A discussion of the scientific recommendations which were made to address these concerns

If an archived advisory committee meeting webcast is used, include the specific video link and time signature to which you are referring in brackets in the body of the paper.

### Resources

- Committees, meetings, and related materials can be found at: <https://www.fda.gov/AdvisoryCommittees/CommitteesMeetingMaterials/default.htm>
- FDA’s Information Repository for Academic Institutions (FDA IRAI), containing guidances and other resources, can be found at: [www.irai-online.org](http://www.irai-online.org)
- FDA Strategic Plan: Advancing Regulatory Science can be found at: <https://www.fda.gov/downloads/scienceresearch/specialtopics/regulatoryscience/ucm268225.pdf>

**Summer 2017**

**CTSC 5025: Grading Rubric for Paper Proposal**

|  | **5-Exemplary** | **3-Sufficient** | **0-Not Evident** |
| --- | --- | --- | --- |
| **Identifies a product or technology** | - Clearly identifies the product or technology of interest and provides 1-2 sentences detailing indication and use | - Identifies the product or technology of interest, but may not sentences background on indication and use | - Does not identify a specific product or technology |
| **Defines associated safety, efficacy, and quality concerns** | - Lists 3 items of concern associated with safety, efficacy, and/or quality | - Lists 1-2 items of concern associated with safety, efficacy, and/or quality | - Does not mention any items of concern associated with safety, efficacy, and/or quality |
| **Identifies a relevant clinical trial or guidance associated with the product or technology (note that trials may be in progress and guidances may be in draft form at the time of the proposal)** | - Identifies a clinical trial or guidance relevant to the technology or product selected - Includes 1-2 sentences highlighting the regulatory science connecting the trial and/or guidance with the product or technology | - Identifies a clinical trial or guidance relevant to the technology or product selected | - Does not identify a clinical trial or guidance relevant to the technology or product selected |
| **Identifies a regulatory science priority area related to the technology or product** | - Specifically names a priority area from the FDA Advancing Regulatory Science document - Explains how the technology or product is related to the priority area in 2-3 sentences | - Specifically names a priority area from the FDA Advancing Regulatory Science document | - Does not name a priority area from the FDA Advancing Regulatory Science document |
| **Paper proposal properly attributes citations** | - Paper includes citations and complete bibliography in JAMA format | - Paper includes some citations - Bibliography not complete or properly formatted | - Paper does not include citations or bibliography |

**Summer 2017**

**CTSC 5025: Grading Rubric for Final Paper**

|  | **5-Exemplary** | **3-Sufficient** | **0-Not Evident** |
| --- | --- | --- | --- |
| **Identifies a product or technology and defines associated safety, efficacy, and quality concerns** | - Clearly identifies the product or technology of interest and provides brief background on indication and use - Outlines the safety, efficacy, and quality concerns associated with the product | - Identifies the product or technology of interest, but may not provide background on indication and use - Outlines some of the safety, efficacy, and quality concerns associated with the product | - States only the name of the product or technology - Does not outline the safety, efficacy, and quality concerns associated with the case of choice |
| **Discusses any preclinical studies and clinical trials, including results published prior to approval of product (or, in the case of a technology, passage of related guidance[s])** | - Identifies relevant preclinical studies and clinical trials associated with the case - Discusses key characteristics of these investigations, focusing on study subject selection, methodology and outcome measures - Critiques the study methods and identifies areas in which novel techniques for assessment of safety, efficacy, and quality were applied | - Identifies some of the relevant preclinical studies and clinical trials associated with the case - Discusses some characteristics of these investigations - Identifies some areas in which novel techniques for assessment of safety, efficacy, and quality were applied | - May identify some of the relevant preclinical studies and clinical trials associated with the case, but does not discuss in detail |
| **Includes discussion of relevant guidances and regulations, or why they are needed (if not yet created)** | - Specifically cites relevant guidances and regulations (draft or final), discusses which scientific components are related to assessment of the case study’s safety, quality, and efficacy concerns - If no guidances available addressing one or more of the concerns, then proposes the gap as a potential area for future development | - Mentions some relevant guidances and regulations - Discusses a few of the scientific components are related to assessment of the case study’s safety, quality, and efficacy concerns - May or may not identify gaps and propose them as areas for future development | - Alludes to a guidance or regulation but does not specifically name it - Does not discuss the scientific components are related to assessment of the case study’s safety, quality, and efficacy concerns - Does not identify gaps and propose them as areas for future development |
| **Discussion of the new tools, standards, and/or recommendations needed to evaluate the safety, efficacy, and quality of product or technology** | - Gives a detailed overview of the tools, techniques, standards, and/or recommendations used to evaluate the product or technology - If these standards are not yet established or have not been created, outlines potential areas for testing - Addresses safety, efficacy, and quality - Identifies and discusses relevant priority areas of regulatory science, as defined by the FDA | - Gives a brief or high-level overview of the tools, techniques, standards, and/or recommendations used to evaluate the product or technology - May or may not include proposed testing or issues for consideration if standards do not yet exist - May or may not address safety, efficacy, and/or quality - May or may not identify relevant priority areas of regulatory science, as defined by the FDA | - Does not discuss the tools, standards, techniques, standards, and/or recommendations needed to evaluate the safety, efficacy, and quality of the product - Does not reference FDA regulatory science priority areas |
| **Summarizes take-home points in conclusion** | - Clearly and concisely summarizes main points of paper - Includes summary of key safety, efficacy, and quality concerns with the case and how those might be or were addressed | - Summarizes main points of paper - May or may not include summary of some of safety, efficacy, and quality concerns, and some solutions | - Does not include a summary of main points of paper - Does not address safety, efficacy, and quality issues |
| **Paper shows use of primary literature and relevant sources (including regulations and guidances); properly attributes citations** | - Paper includes citations and complete bibliography in JAMA format | - Paper includes some citations - Bibliography not complete or properly formatted | - Paper does not include citations or bibliography |
